# Supplementary material for: Phenotypic defects from the expression of wild-type and pathogenic TATA-binding proteins in new Drosophila models of Spinocerebellar Ataxia Type 17
Source: G3 (Bethesda). 2023 Aug 8;13(10):jkad180. doi: 10.1093/g3journal/jkad180 (PMC10542169; doi:10.1093/g3journal/jkad180)
Supplement: jkad180_Supplementary_Data [file jkad180_supplementary_data.zip › Supplemental_Figure_Legends_G3-2023-404402.docx]

**SUPPLEMENTAL MATERIAL**

**Supplemental Figure Legends**

**Figure S1: Impact of aging on TBP aggregation in male flies.** Representative Western blots in uninduced male control (RU- (OFF)) and experimental flies (RU+ (ON)) with adult-specific pathogenic (Q63, red) or wild-type (Q25, black) TBP expression in weeks 1, 3, and 5 of adulthood, indicated by lane. Expression was induced on adult day 3 in **(A)** all tissues **(quantified in D)**, neurons **(B, E)**, or glia **(C, F)** and continued until sample isolation. Black arrows: TBP, Red arrows: polyQ-expanded TBP, Blue brackets: SDS-resistant TBP, asterisks: non-specific signal. Quantification and statistics: Kruskal-Wallis test with Dunn’s post-hoc comparison. Bars indicate mean -/+ SD, n=5 biological replicates of 3 flies per lysate.

**Figure S2: Statistical comparisons for all groups shown in Figures 6-8.** Combined longevity (A, C, E) and motility (B, D, F) comparisons for flies expressing TBP ubiquitously throughout adulthood, (A, B), in adult neurons (C, D), and adult glia (E, F). Graphs are re-plots of repetitions shown in figures 6, 7, and 8. Statistics: Survival: log-rank, climbing speed: linear regression. Longevity and motility experiments performed in 2 biological replicates, n≥173.
