# Supplementary figures and images for: Phenotypic defects from the expression of wild-type and pathogenic TATA-binding proteins in new Drosophila models of Spinocerebellar Ataxia Type 17
Source: G3 (Bethesda). 2023 Aug 8;13(10):jkad180. doi: 10.1093/g3journal/jkad180 (PMC10542169; doi:10.1093/g3journal/jkad180)

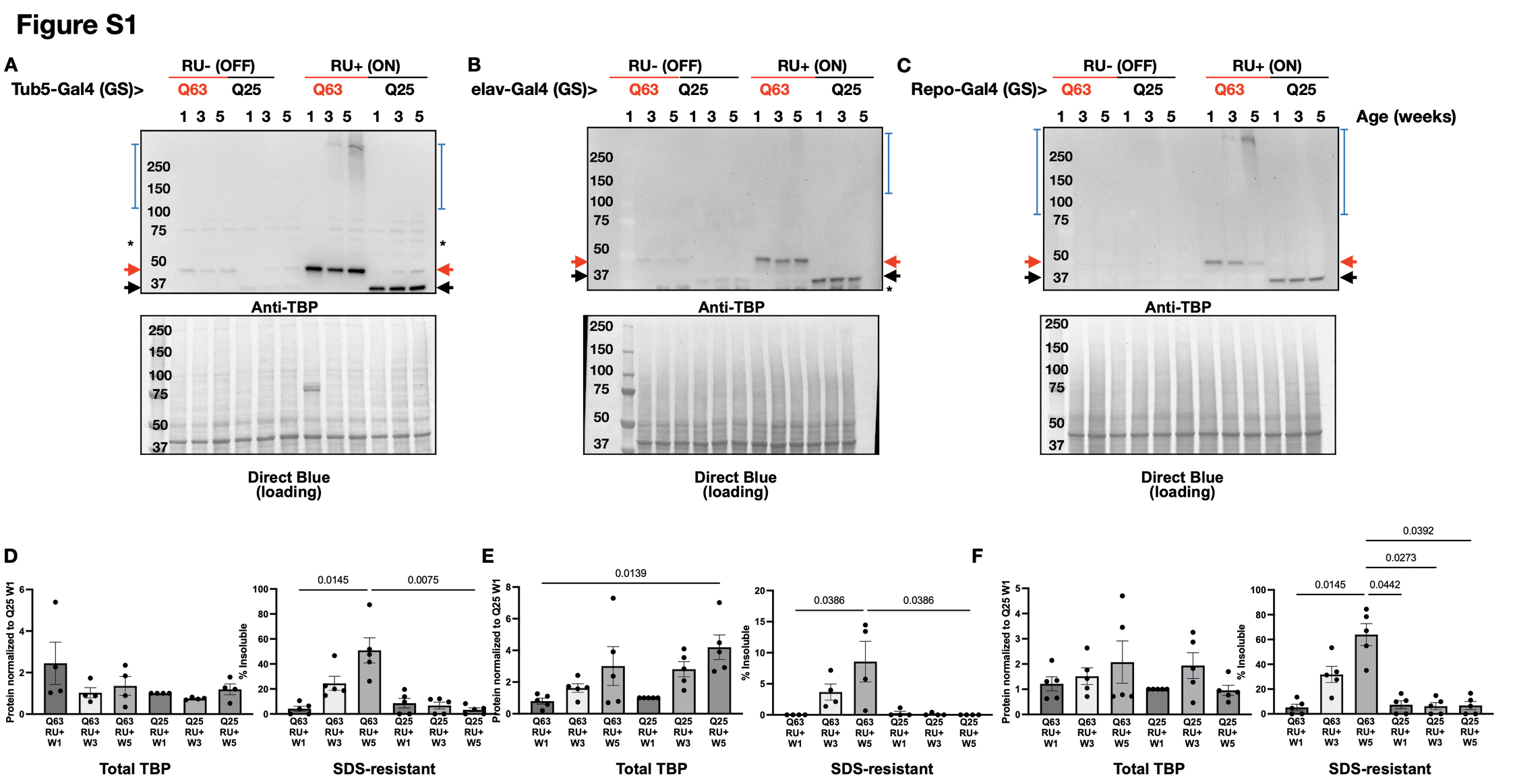

Supplement: jkad180_Supplementary_Data [file jkad180_supplementary_data.zip › Figure_S1_G3-2023-404402.tif]

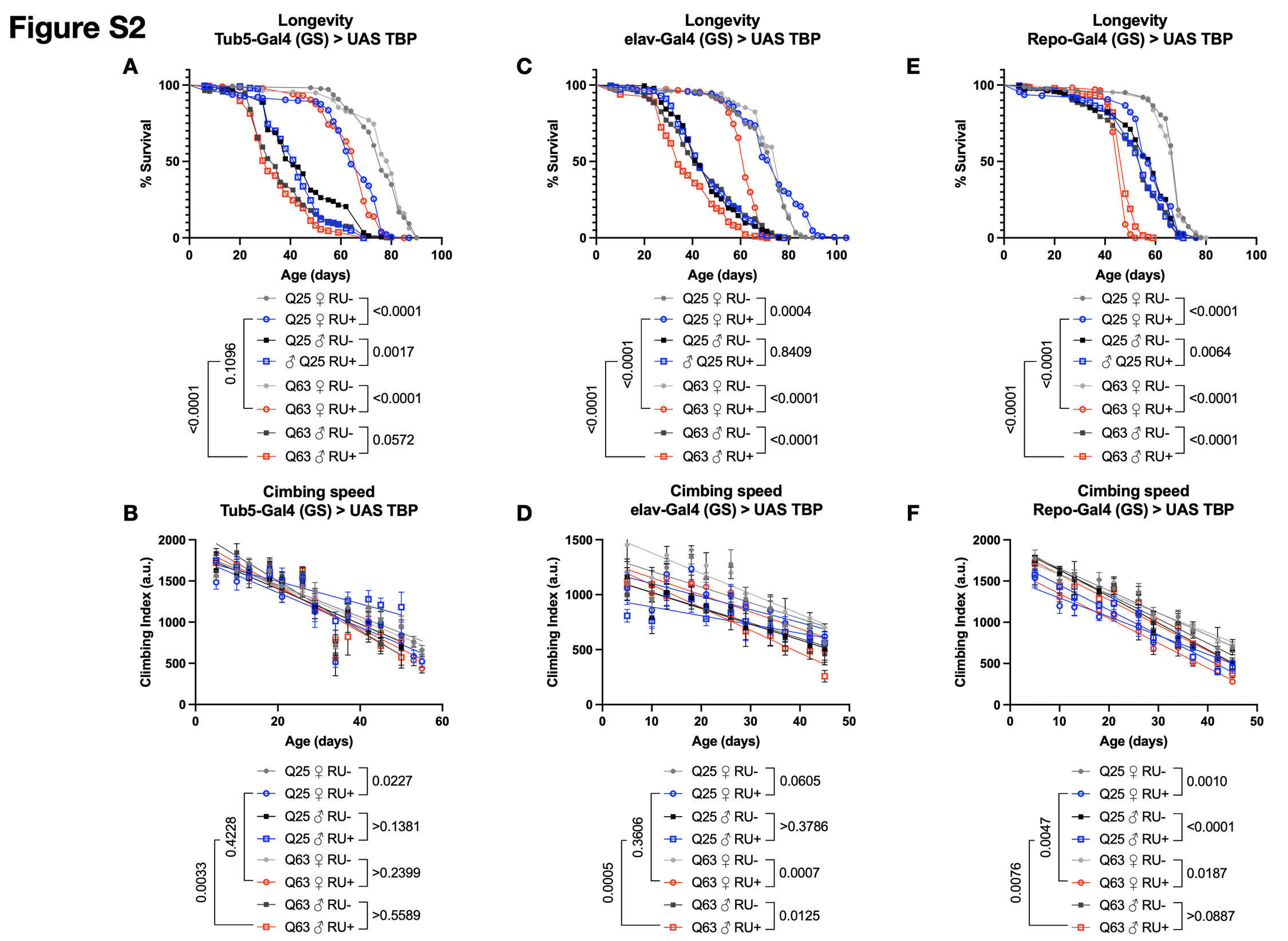

Supplement: jkad180_Supplementary_Data [file jkad180_supplementary_data.zip › Figure_S2_G3-2023-404402.tif]
